# Periodontists’ perceptions and attitudes toward the use of social media for professional purposes in Saudi Arabia

**DOI:** 10.1186/s12903-023-03444-7

**Published:** 2023-10-08

**Authors:** Banna Alnufaiy, Hanadi Ghurmallah Alzahrani, Abdullah Saad Alqahtani, Khalid Gufran, Alfaisal Alhamdan, Khalid Alhamdan

**Affiliations:** 1 Department of Preventive Dental Sciences, College of Dentistry, Prince Sattam Bin Abdulaziz University, 11942 Alkharj, Saudi Arabia; 2 Internship, college of dentistry, Gulf Medical University, Ajman, United Arab Emirates; 3 Department of Periodontics and Community Dentistry; College of Dentistry, King Saud University, KSU Unite-1 /12372, PO Box;7669, Riyadh, Saudi Arabia

**Keywords:** Social media, Promotion, Periodontist, Dentist, Saudi Arabia

## Abstract

**Background:**

There is a notable increase in the usage of social media platforms, especially for health communication, as more clinicians and patients count on this kind of technology. Therefore, this research aimed to investigate the perception and attitude toward social media (SM) use for professionalism and dental practice promotion among periodontal specialists working in Saudi Arabia (KSA).

**Methods:**

Electronic surveys were distributed in person or online using WhatsApp, Snapchat or Email from October 2022 until March 2023. The questionnaire was targeting periodontists in KSA, and it consists of three parts: the first part includes sociodemographic and professional data; the second part asks about the daily usage of SM in dental practice; and the third part asks about the periodontist's opinion about SM usage. Descriptive data were presented as numbers and percentages. The association between the demographic variables and the means of periodontists’ opinions was tested using Pearson’s chi-square test. Any value equal to or less than 0.05 was considered statistically significant.

**Results:**

A total of 121 responses were received from the periodontists. Most of the participants were in the 36–45 age group, with a percentage of 40.5%. In terms of gender, males made up 52.9% of the population, while females made up 47.1%, and the majority of the participants were consultants. The most common SM platform used by periodontists for daily using and dental practice promotion was Snapchat (56.2%), followed by Instagram and Twitter (54.5% and 49.6%, respectively). There was a significant difference in proportions among Twitter, Snapchat and TikTok between older and younger periodontists, with a *p* value < 0.05. There was no significant difference between the gender of the participants and their opinions regarding the usage of SM.

**Conclusions:**

This study highlighted the effectiveness of SM in the promotion of dental practices and the discipline of periodontics, as more clinicians and patients rely on this kind of technology. These online platforms can improve periodontal practice in terms of dental health education, counseling, advertising, and oral health services.

## Background

Social media (SM) consists of online networking sites that enable people to build relationships or share similar interests, photos and activities. Currently, there are various SM platforms, including Twitter, Facebook, Snapchat, Instagram, WhatsApp, and YouTube [1]. Meanwhile, the community is largely dependent on these online platforms to locate available services such as business, marketing, education and advertising. Due to the increase in technological advancement, the number of internet network users in Saudi Arabia at the beginning of 2018 increased to 30 million, with penetration that reached 91% in the country [2, 3]. Although the main purpose of SM is to share daily activity, today, many are using this tool to look for medical advice and specific centers and to communicate with health care providers [4]. For that reason, SM has become a vital tool not only for patients but also for health care providers because it helps enhance the process of learning and professional education in both dental and medical fields [5, 6]. For example, practitioners in the dental field find it helpful to exchange presentations and educational videos showing different techniques and dental procedures from which other dentists or dental students can benefit [7]. In addition, students have the chance to discuss various case scenarios, diagnoses and share opinions through virtual sessions [8]. According to a previous study in 2019, 76% of participants agreed that SM can enhance the skills and knowledge of different students in the medical field in Saudi Arabia [4]. Another study showed that some students have been accessing YouTube to watch videos of specific dental procedures before they actually perform it [6, 9]. It seems that students generally use YouTube and Instagram to enhance their educational skills while using Twitter to stay in touch and communicate with instructors [10, 11].

According to a recent study conducted in Saudi Arabia, patients attending both private and government hospitals were using SM for communication and entertainment purposes. Additionally, research revealed that Snapchat (71.1%) and Instagram (66.9%) were the two platforms that patients most frequently used [12]. In conjunction, this was supported by another study, which showed that SM can improve the method of communication between two parties, resulting in improved patient experiences in terms of satisfaction, motivation and knowledge [13]. Additionally, it has been stated that patients tend to remember information that they have attained from websites more than that from other resources [14]. Based on reports, most Saudi Arabian participants preferred getting their health-related information from trusted official sources [12, 15].

During the COVID-19 pandemic, social media networks have been helpful in assisting individuals to stay connected. Doctors and dentists noticed an increase in social media activity during the pandemic [16, 17]. For the effective communication of current knowledge on the novel coronavirus SARS-CoV-2 and to stay up with evolving best practices, many in these two professional organizations have grown to depend more and more on social media [18]. A study by Kwok et al. (2020), found that in the Hong Kong community, SM (Internet and WeChat) was considered a primary information source for COVID-19 [19]. For that reason, many medical and dental professionals used social media during the global pandemic for information exchange, professional networking, interaction with the public and patients, as well as for training and educational purposes [16, 20].

Dental professionals can engage and connect with many individuals on social media, which offers a great platform for communication. Social media opens up opportunities for dental professionals to educate and inform their audience about oral health [12, 21]. Dentists can spread awareness about the value of preventative care, the advantages of routine checkups, and the necessity of oral hygiene by publishing educational articles, videos, infographics, and recommendations on social media platforms [22–24]. The information it provides offers individuals the resources they need to take control of their oral health, which improves overall wellbeing.

However, there has been a great diversity of opinions regarding the benefits of using SM in dental practice. The biggest concern were security and legal problems [25–27]. As the dental care providers may have issues managing their professional image and relationship with patients because everyone could access rapidly any information posted online [28]. Moreover, the practitioners and patients may face consequences that threaten their privacy as a result of using these platforms [29]. Previous report stated that dentist do not understand process, methods and concepts related to SM communication [30]. In addition to that, counts of online papers of medical literature have been labeled as low quality, which if spread into the wrong way, could lead to lethal issues such as the overdose of drug use or unnecessary cosmetic procedures [21, 31]. In spite of that, we need to highlight a clear guidelines to improve the dentists’ skills in such field professionally and safely. Furthermore, there is still no clarity about the effect of SM on periodontal practice and this area is still vague for both patients and periodontists. Additionally, to our knowledge, no studies have linked SM and periodontics in Saudi Arabia. In this sense, the outcomes of this study will help in developing programs and workshops that will be directed to enhance dental practice, knowledge and patient-doctor interaction among periodontists in Saudi Arabia. Based on that, this research aimed to investigate the perception and attitude toward the SM use for professionalism and dental practice promotion among periodontal specialists working in Saudi Arabia.

## Methods

### Ethical approval

The standing committee of bioethics research (SBCR) of Prince Sattam bin Abdulaziz University approved the study protocol (SCBR-074–2022). Moreover, the study was conducted according to the guidelines of the Declaration of Helsinki.

### Study design

This cross-sectional and questionnaire-based study was conducted on a sample of periodontists working in Saudi Arabia. A convenience sample was selected from database of Saudi Society of periodontology. Study subjects were invited to participate in this study voluntarily in person or online using WhatsApp, Snapchat or Email.

### Study sample

The sample size was calculated based on a 95% confidence level, a 5% margin of error, and a 30% response distribution. According to sample size software (http://www.raoso ft.com/samplesize.html) [32], the minimum needed sample size was 120 for the periodontist.

### Study instrument

To ensure test–retest reliability, the survey was given to a pilot group that consisted of 10 individuals before distribution. Periodontists and postgraduate dental students were answered the questionnaire which was sent through WhatsApp. Then, the pilot group repeated the survey again after one week to ensure the clarity and acceptability of the questions. As a result, minor changes were made by rephrasing two questions to prevent any confusion. Also, one of them suggested to design it in more flexible way, in which the participant can move back and forth between the pages to review and change their answers before submitting.

### Data collection

The survey was adapted from previous similar studies on the use of SM by medical professionals according to CHERRY’s Check-list and was basically a closed ended questionnaire [4, 15]. The questionnaire was prefaced with an introductory paragraph to clarify the objective of the study, voluntary participation and about the length of time it take to complete the survey ( 2 min). Informed consent was obtained from all participants who agreed to participate in this study. The study was explained and ensured confidentiality for all the participants. The questionnaire consisted of three parts: the first part includes sociodemographic and professional data such as age, gender, work experience and qualification. The second part asks about the daily usage of SM in dental practice; and the third part asks about the periodontist's opinion about SM usage ( Agree, not sure, disagree).

### Statistical analysis

Microsoft Excel (2021) version was used to enter the data, and the sheets were transferred to Statistics IBM SPSS for Mac, Version 22 for statistical analysis (IBM Corp., Armonk, NY, USA). Descriptive data were presented as numbers and percentages. The association between the demographic variables and the means of periodontists’ opinions was tested using Pearson’s chi-square test. Any value equal to or less than 0.05 was considered statistically significant.

## Results

**Fig. 1:**
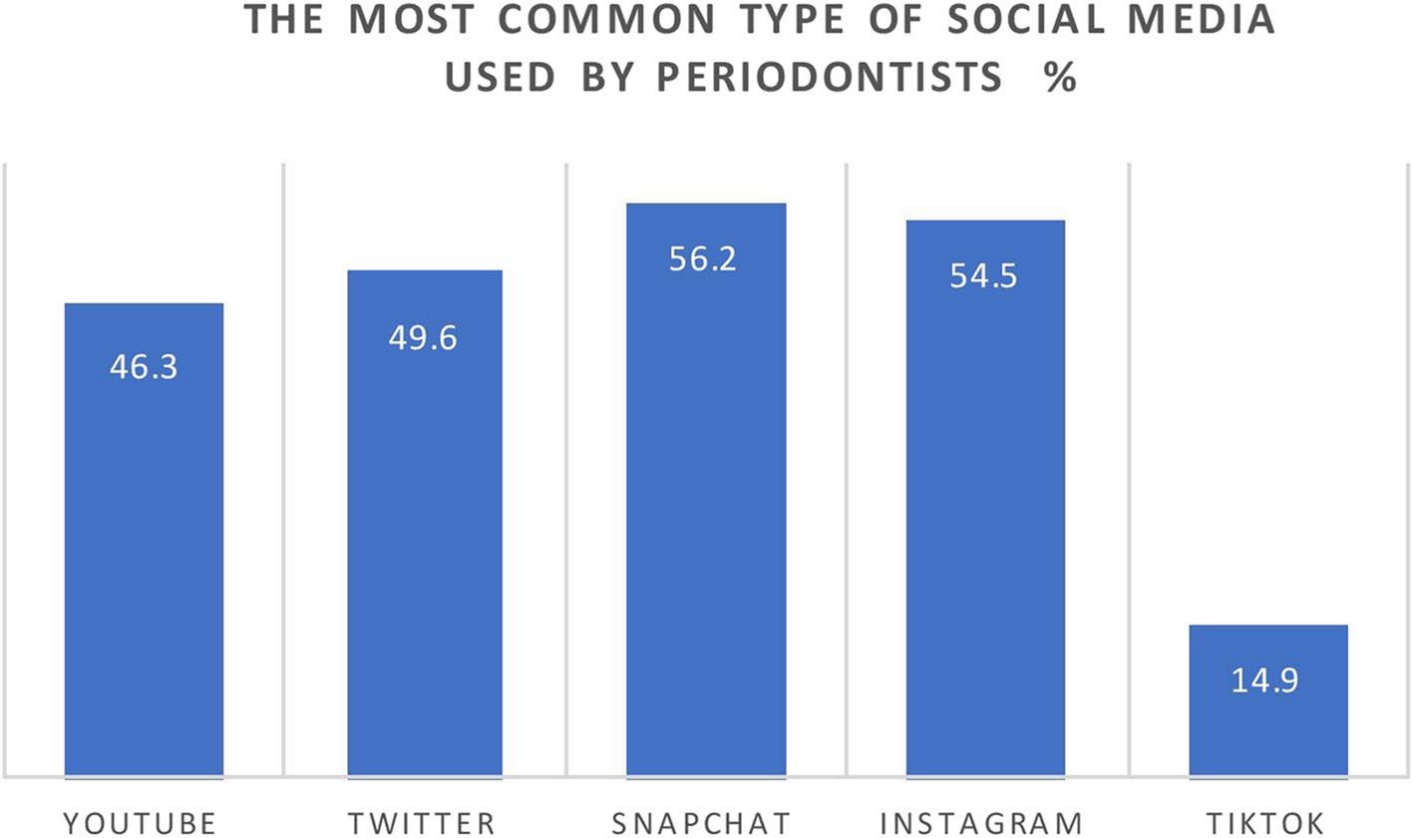
The most common type of social media used

**Fig. 2:**
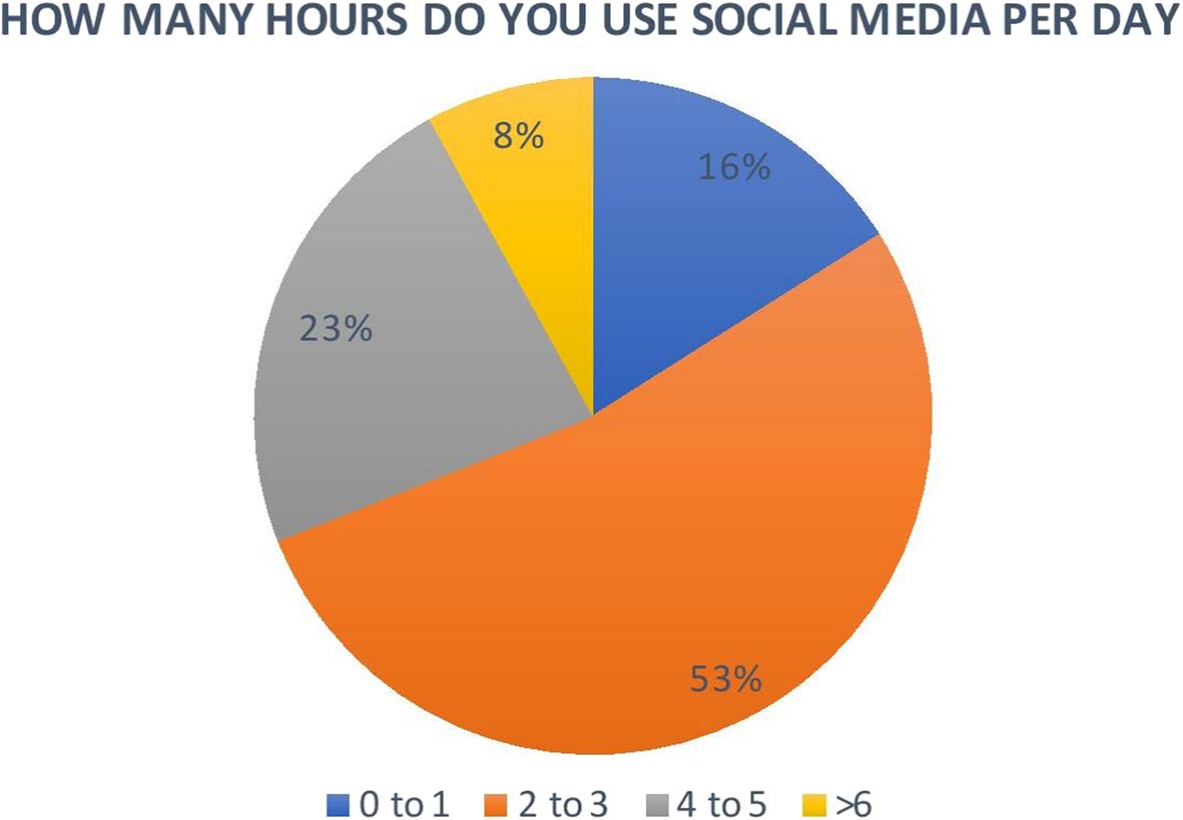
The daily use of social media (in h)

From October 2022 until March 2023, 300 surveys were sent out and a total of 121 responses were returned. An emphasis email or message was sent on a weekly basis as means of a reminder for non-respondent periodontists. The response rate was 40.3%. Out of the 121 contributors, 119 responded with the completed survey (the completion rate of the survey was 98.3%). The sociodemographic data of the participants are shown in Table 1. Most of the participants were from the age group of 36–45 with a percentage of 40.5%. This was followed by the age group of 26–35 (38.8%), while only 20.6% were > 45 years of age. Regarding gender, the participants in our study were 52.9% males compared to 47.1% females (*N* = 64 and 57, respectively). The majority of the participants (44.6%) were from the central region of the KSA, and only 9.15% worked in the north of the country. More than half of the periodontists were Saudi (72.7%), and less than a third of them (25.6%) were non-Saudi. In addition, the majority of the participants (47.1%) were consultants. In addition, the study included specialists and residents (34.7% and 12.4%, respectively). Most of the participants had more than 10 years of experience (43.0%), and only 5.8% had less than 2 years. The most common SM platform used by periodontists was Snapchat (56.2%), followed by Instagram and Twitter (54.5% and 49.6%, respectively) (Fig. 1). Approximately more than half of the periodontists spent 2–3 h daily for different purposes (Fig. 2). At the same time, 23.1% of the participants stated that their website is the main communication tool for patients to access in their dental practice. Regarding dental content, the most common type of content the participants posted on SM were pictures of before and after cases. In addition, the majority of periodontists (47.9%) think that the content of the profile is the most attractive thing to patients in comparison to the complexity of the treatment or the number of followers ( Table 2).

**Table 1:** Demographic data

Variables	Level	n	%
Gender (*n* = 121)	Male	64	52.9
	Female	57	47.1
Age (*n* = 121)	26–35	47	38.8
	36–45	49	40.5
	> 45	25	20.6
Nationality (*n* = 119)	Saudi	88	72.7
	Non-Saudi	31	25.6
Area of Work (*n* = 121)	Center	54	44.6
	East	16	13.2
	North	11	9.1
	West	22	18.2
	South	18	14.9
Years of experience (*n* = 119)	0–2	7	5.8
	3–5	26	21.5
	6–10	34	28.1
	> 10	52	43.0
Position (*n* = 120)	Consultant	57	47.1
	Specialist	42	34.7
	Resident	15	12.4
	Others	6	5.0

**Table 2:** Practice related to social media usage

Practice related to social media usage	Type	n	%
Which type of online media does your workplace currently have for patients to access?	Website	28	23.1
	Text messages	22	18.2
	Instagram	26	21.5
	Facebook	2	1.7
	Twitter	18	14.9
	Others	20	16.5
The most common types of dental content you posted on social media	Pictures of before and after cases	38	31.4
	General information for public health	69	57.0
	New techniques and methods	22	18.2
What, in your opinion, is more attractive to patient?	The complexity of the treatment that have been posted	33	27.3
	No. of followers	29	24.0
	The content of the profile	58	47.9

**Fig. 3:**
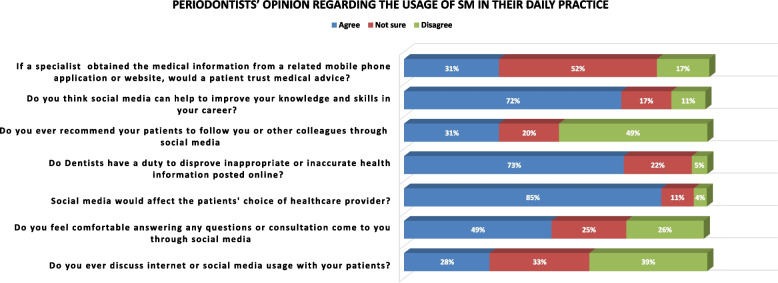
Periodontists’ opinion regards the usage of social media in their daily practice

Periodontists’ opinions regarding the usage of SM in their daily practice are presented in Fig. 3. The findings revealed that 31% of the participants agreed that a patient would trust medical advice when a specialist provided the medical information on a mobile application, while 52% were not sure about that. Furthermore, 72% of the periodontists agreed that SM could help improve their knowledge and skills in their dental careers. Moreover, the majority of the participants believed that they had a responsibility as care providers to correct any inaccurate health information posted online. At the same time, only 26% of specialists felt uncomfortable answering consultations through SM. A total of 85% of periodontists agreed that SM is an effective tool for influencing patients' choices of dental care providers. Table 3 presents the relationship between the participants’ age and the type of SM use. The findings indicated that younger care providers mostly used Snapchat, followed by Instagram, compared to older care providers. There was a significant difference in proportions among Twitter, Snapchat, and TikTok between older and younger periodontists, with a *p* value < 0.05. On the other hand, there was no significant difference between the gender of the participants and their opinions regarding the usage of SM ( Table 4).

**Table 3:** The association between respondents’ age and the type of social media usage

Type of social media	Age in years			*p* value*
	26–35 n (%)	36–45 n (%)	> 45 n (%)	
YouTube	20 (35.7)	23 (41.1)	13(23.2)	0.115
Twitter	24 (40.0)	27 (45.0)	9 (15.0)	0.003*
Snapchat	35(52.2)	28 (41.8)	4 (6.0)	0.000*
Instagram	27(40.9)	26 (39.4)	13(19.7)	0.008
TikTok	7 (38.9)	8 (44.4)	3 (16.7)	0.000*

*Significant at *p* ≤ 0.05

**Table 4:** The association between respondents’ gender and their opinions regarding the usage of SM

opinions regarding the usage of SM	Gender	Agree		Not sure		Disagree		*p* value*
		**n**	**%**	**n**	**%**	**n**	**%**	
Do you ever discuss internet or social media usage with your patients?	Male	16	26.2%	23	37.7%	22	36.1%	**0.664**
	Female	17	29.8%	17	29.8%	23	40.4%	
Do you feel comfortable answering any questions or consultation come to you through social media	Male	33	53.2%	14	22.6%	15	24.2%	**0.686**
	Female	26	45.6%	16	28.1%	15	26.3%	
Social media would affect the patients' choice of healthcare provider?	Male	55	88.7%	5	8.1%	2	3.2%	**0.657**
	Female	48	85.7%	7	12.5%	1	1.8%	
Do Dentists have a duty to disprove inappropriate or inaccurate health information posted online?	Male	46	74.2%	12	19.4%	4	6.5%	**0.424**
	Female	41	74.5%	13	23.6%	1	1.8%	
Do you ever recommend your patients to follow you or other colleagues through social media	Male	21	33.9%	12	19.4%	29	46.8%	**0.943**
	Female	17	30.9%	11	20.0%	27	49.1%	
Do you think social media can help to improve your knowledge and skills in your career?	Male	48	77.4%	10	16.1%	4	6.5%	**0.167**
	Female	38	66.7%	9	15.8%	10	17.5%	
If a specialist obtained the medical information from a related mobile phone application or website, would a patient trust medical advice?	Male	24	38.7%	27	43.5%	11	17.7%	**0.116**
	Female	13	22.8%	35	61.4%	9	15.8%	
What in your opinion is more attracting to the patient?	Male	15	24.2%	31	50.0%	16	25.8%	**0.945**
	Female	13	23.2%	27	48.2%	16	28.6%	

*Significant at *p* ≤ 0.05

## Discussion

SM considered as one of the most popular web-based activities, which had an estimated 2.9 billion members as of 2019 and is expected to reach 3.4 billion by 2023 [9]. SM has become a powerful tool that can impact our communication, connection with others, sharing information, and exploring available services, which including dental services [33, 34]. To our knowledge, this study is the first to investigate the perception of SM use for professionalism and dental practice promotion among periodontal specialists working in Saudi Arabia.

The results of this study revealed that most of the participants were from the age group of 36–45, with a percentage of 40.5%, which is in agreement with previous reports that younger-aged practitioners were more frequent users of social platforms than older dentists [21, 34, 35]. However, only 20.6% of the participants in our study were aged over 45. This finding is consistent with another study, which found that only 3% of expert dental practitioners with 15 years of experience in the field responded [7]. This low percentage may be because skilled dentists have established patient relationships and do not need to advertise their services on SM. Despite this, 65% of physicians in a 2013 survey of more than 4000 said they used SM for professional reasons [9]. In addition, in this study, 85% of periodontists agreed that SM is an effective tool for influencing patients' choices of dental care providers and this percentage is very close to Saudi physicians population who believed the same [4].

At the same time, 72% of the periodontists agreed that SM could help improve their knowledge and skills in their dental careers. This is in consistent with a study done in KSA of more than 750 participants, including dentists and dental students, in which 61.1% believed that SM is a good way to learn and refine future professionalism [10].

In our study, the most common SM platform used by periodontists was Snapchat (56.2%), followed by Instagram and Twitter (54.5% and 49.6%, respectively). That could be explained by the chronology of the emergence of these applications, as Twitter is the oldest and Snapchat is the most recent. Moreover, there was a significant difference in proportions among Twitter, Snapchat and TikTok between older and younger periodontists, with a *p* value < 0.05. These findings indicated that younger care providers mostly use Snapchat followed by Instagram. In contrast, forty-nine percent of the respondents in another report for dentists, in the same region, reported that Instagram was the most powerful platform for advertising [7].

Instagram, a major SM platform that is a significant source of health information, has grown in popularity in recent years [36]. Users of this site can post photos and videos and engage with user-generated content by using hashtags. It is also the most widely used social networking platform among people between the ages of 15 and 25 [37]. The number of articles in PubMed that related to health literature, employed Instagram as part of the study methodology, and examined the influence of this SM on medical education increased by 89% from 2019 to 2020. By 2021, as many publications as everything before 2020 had been indexed in this database [37, 38]. However, a survey performed online by the Levin Group Data Center between July and November 2015 revealed that Facebook was the most popular platform for dentists, with almost 88.8% of them using it to advertise their services, but at the same time, in this study, 23.1% of the participants stated that their website is the main communication tool for patients to access in their dental practice [39]. With a user share of 87.4% in Saudi Arabia in 2022, WhatsApp was the most commonly used SM platform nationwide. Approximately 29.5 million people used SM in Saudi Arabia as an entire nation that year [40]. These details could help dentists decide which platform has the biggest influence in their area and is therefore the best match for their needs.

In this study, 85% of periodontists agreed that SM is an effective tool in patients’ choice of dental care provider. This is in agreement with other reports that SM marketing is more useful to dentists than other traditional advertising methods [7, 12]. Furthermore, this can shed light on the importance of this online technology. As it grows rapidly among the population, there is a great opportunity to recruit patients into dental clinics in Saudi Arabia [23, 41].

Periodontists must understand that daily use of SM platforms should be determined by the type of SM frequently used by their patients. Moreover, the everyday usage of SM was alarming since our study revealed that more than half of the periodontists spent 2–3 h daily for different purposes, which might indicate signs of addiction to social networking sites [42].

The most common type of dental content the participants posted on SM was general information for public health. In addition, many periodontists think that the content of the profile is the most attractive to patients (47.9%) in comparison to the complexity of the treatment or the number of followers. These findings are in accordance with those of a previous study in which they stated that the most common type of dental content posted was general information that was useful for patients (34%) [7]. Moreover, according to a study conducted in 2012, 37% of dentists in the US used SM to gather information from dental and health care providers [43].

However, improper SM use could reveal dental practitioners' inadequate professionalism. This is supported by the findings of a previous study on general dentists, which found that 86% of respondents who posted patient photos did so without receiving the patients' verbal or written informed consent, with 65% of respondents presuming that the patients would not recognize themselves in the photos [7, 23].

In accordance with other reports, the greatest number of the participants in our study believed that they have a responsibility as care providers to correct any inaccurate health information posted online [21, 44]. This is due to the awareness and responsibility of health care providers, who always feel that they have a duty to validate and control any medical or dental information posted online [2, 45]. At the same time, only 26% of specialists were uncomfortable answering consultations through SM. This may be related to the fact that they felt such consultation would be inappropriate due to the lack of information related to the cases or it could be due to their fear of legal consequences. There are some limitations of this study, the most noticeable one was the small sample size of participants and the responded of the target group. There is a good proportion of periodontists population in Saudi Arabia which either do not like to react to online surveys or use such communication methods. In addition to that, some of them lack to the prober dealing with a survey in which they accepted to fill out voluntary but skipped some questions. Also, the possible other limitation could be related to the majority of the sample which being mainly from central and west regions. The third limitation was the lack of a probability solid sampling technique. Although, the sampling was according to the database of Saudi Society of periodontology, this may affect the generalizability of the results to the whole periodontists in Saudi population. Future studies with a bigger sample size are recommended with good design cluster sampling which includes different geographic parts of Saudi Arabia. Also, it would be interesting to know if there is a different opinion between periodontists in private and government section in Saudi Arabia.

## Conclusions

This study highlights that most of periodontists agreed that SM is an effective tool for influencing patients' choices, which affect their periodontics and dental practice promotion specially in younger age practitioners. As more clinicians and patients rely on this technology, the use of these online platforms can enhance periodontal practice in terms of oral health services, advertising, counseling, and dental health education. The use of SM can significantly expand the reach and impact of periodontists' work and enhance patients' health, but those who choose to use it should be aware of the potential hazards and issues that they could encounter.

## Data Availability

The datasets used and analyzed during the current study are available from the corresponding author on reasonable request.

## Abbreviation

SM: Social Media
